# Osteitis of the radius following Bacillus Calmette–Guérin vaccination at birth: a case report

**DOI:** 10.1186/s13256-017-1446-5

**Published:** 2017-10-04

**Authors:** Abdelmoneim E. M. Kheir, Salah A. Ibrahim, Azza Abdelsatir, Mustafa E. Bahar

**Affiliations:** 1 Department of Paediatrics and Child Health, Faculty of Medicine, University of Khartoum, Khartoum, Sudan; 2 Department of Pathology, State Ministry of Health, Khartoum, Sudan; 3 Department of Radiology, Soba University Hospital, Khartoum, Sudan; 4 Department of Paediatrics and Child Health, Faculty of Medicine, University of Khartoum and Soba University Hospital, P.O. Box 102, Khartoum, Sudan

**Keywords:** Osteitis of radius, Tuberculosis, BCG, Vaccine, Sudan, Case report

## Abstract

**Background:**

The Bacillus Calmette–Guérin vaccine, which is used for the prevention of tuberculosis, is considered protective against the severe forms of childhood tuberculosis. However, some serious adverse reactions including osteitis of the long bones can occur.

**Case presentation:**

We report a case of an 18-month-old Sudanese girl who presented at the age of 3 months with swelling of her left forearm following Bacillus Calmette–Guérin vaccination administered at birth. Radiological and histological investigations confirmed tuberculous osteitis of the distal radius. She responded very well to antituberculous treatment with complete healing at follow-up visits. To the best of our knowledge this is the first case report of osteitis of the radius following Bacillus Calmette–Guérin vaccination described from Sudan.

**Conclusions:**

Bacillus Calmette–Guérin osteitis, although rare, should be considered a possible complication of the Bacillus Calmette–Guérin vaccination, and early diagnosis and treatment are essential.

## Background

The Bacillus Calmette–Guérin (BCG) vaccine, which contains a live attenuated strain of *Mycobacterium bovis*, is considered to be protective against severe forms of childhood tuberculosis including tuberculous meningitis [1]. Although it is a relatively safe vaccine, some serious adverse reactions were reported. The most frequent complications are local subcutaneous abscess or suppurative regional lymphadenitis. Other systemic complications include musculoskeletal lesions, non-fatal disseminated infection, and fatal disseminated disease [2].

Osteitis following BCG vaccination is extremely rare, with an incidence of 0.39/1,000,000, depending on the bacillus used [3]. The lesions are localized to the metaphysis or epiphysis of long bones [4]. Ibrahim *et al*. had reported a case of median nerve compression following BCG vaccination that resolved after drainage of the abscess [5]. There are reports that osteitis following BCG vaccination occurs in places where neonates are vaccinated with MPB70-high-producer BCG substrains. In countries like Finland, Sweden, Czech Republic, and Slovakia, the incidence of osteitis varies, depending on the strain used for vaccination [6]. Most cases of osteitis following BCG vaccination were believed to be caused by hematogenous dissemination of BCG strains due to the abundant blood supply at the time of the initial bacteremia [7].

We report a case of an 18-month-old Sudanese girl who presented at the age of 3 months with swelling of her left forearm following BCG vaccination that was given at birth. Radiological and histological investigations confirmed tuberculous osteitis of the radius. She responded very well to antituberculous treatment with complete healing at follow-up visits. To the best of our knowledge this is the first case report of osteitis of the radius following BCG vaccination described from Sudan.

## Case presentation

**Fig. 1:**
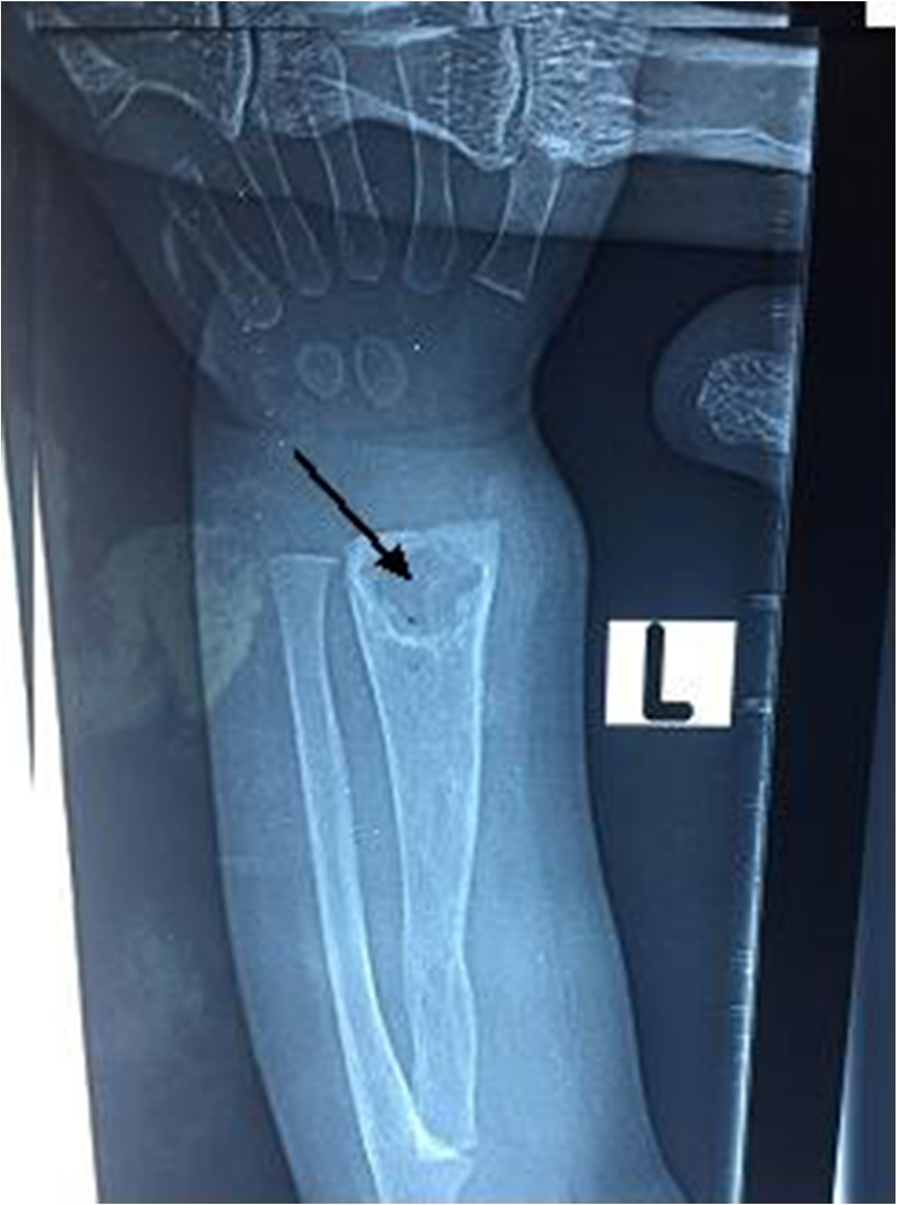
A well-defined metaphyseal lytic lesion with partially sclerotic margin compatible with osteitis (*arrow*)

An 18-month-old Sudanese girl, who had a normal birth history and an uneventful early neonatal period, received the BCG vaccine at birth on her left forearm as part of the routine vaccination schedule in Sudan. There was no history of contact with tuberculosis. At the age of 2 months, she developed a swelling at the BCG site which was tender but there was no discharge; a few weeks later her whole left arm became swollen with reduced movement. There were no lymph nodes enlargements. She was seen at a surgery where an abscess was drained and she was prescribed antibiotics. She continued to be febrile and developed discharging sinus at the site of the wound. Further radiological assessment of her left wrist joint showed a well-defined metaphyseal lytic lesion with partially sclerotic margin and narrow zone of transition not crossing the physis, and soft tissue opacity was noted indicating soft tissue component. These findings were compatible with osteitis (Fig. 1). Her complete blood count was normal, erythrocyte sedimentation rate (ESR) was 5 mm/hour, and her C-reactive protein (CRP) was negative. A chest X-ray (CXR) was also normal.

**Fig. 2:**
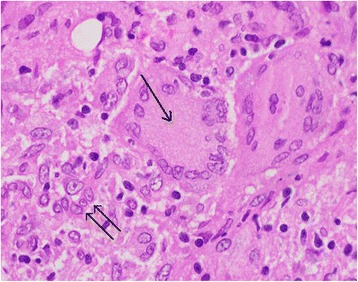
Giant cell granuloma (*single arrow*) surrounded by multiple histiocytes (*double arrow*)

**Fig. 3:**
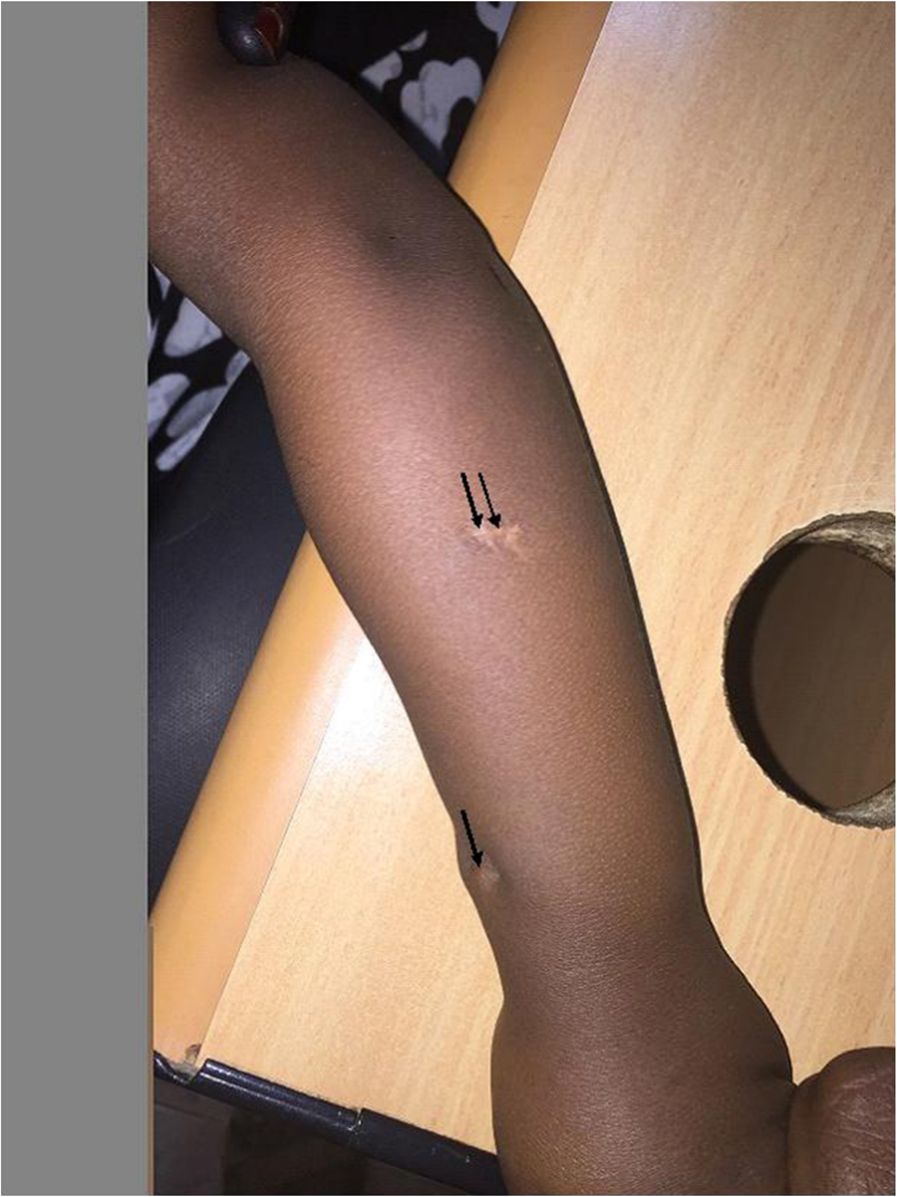
Scar of the healed lesion (*single arrow*) and Bacillus Calmette–Guérin scar (*double arrow*)

**Fig. 4:**
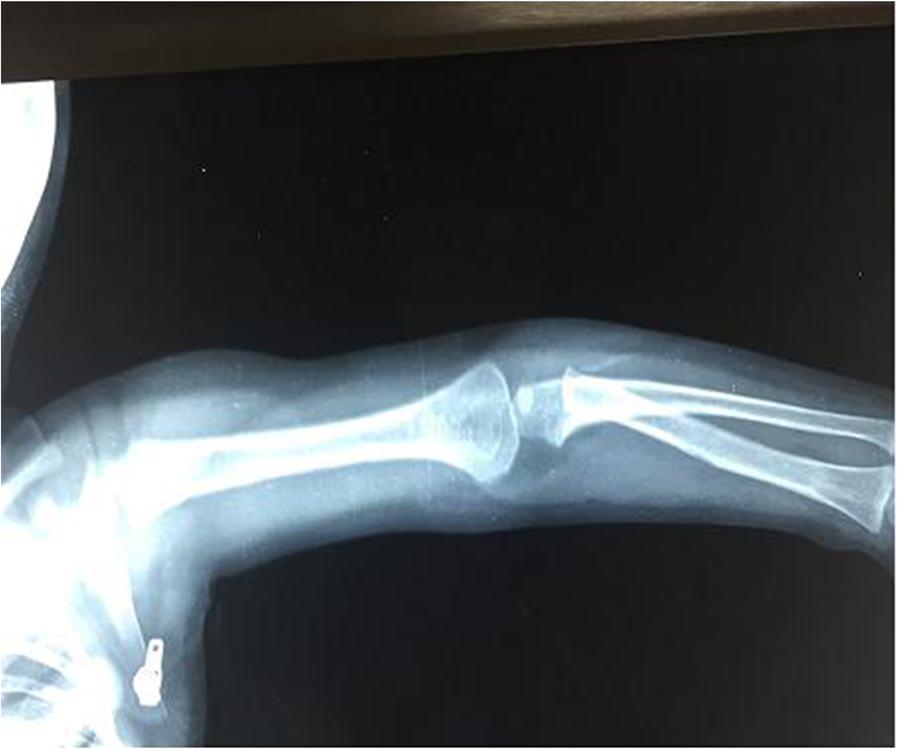
A follow-up X-ray with complete disappearance of the lytic lesion

At this stage the lesion was drained once again and a tissue biopsy showed multiple histiocytes and giant cells with foci of necrosis consistent with tuberculous granuloma (Fig. 2). She was started on antituberculous treatment (isoniazid, rifampicin, and ethambutol). She showed gradual improvement, the swelling subsided, and the wound healed completely; she received the antituberculous treatment for 1 year. At follow-up visits, she used her left forearm normally with full range of movement (Fig. 3). A repeat X-ray showed complete healing (Fig. 4)

## Discussion

The BCG vaccine was named after the two French investigators responsible for its original development in 1923 and has now been used for nearly 100 years in the prevention of tuberculosis [8]. Vitkova and associates studied post BCG vaccination adverse reactions in the period from 1981 to 1993, and demonstrated an elevated incidence of local and regional lymph node complications. In addition, bone and joint involvement following BCG vaccination at birth was reported as a new phenomenon [9].

The baby described here presented with swelling of her left arm following BCG vaccination; this is not surprising as the lesions tend to appear on the same side of the body as the vaccination [10]. Our case presented at the age of 3 months. The age of onset of BCG osteitis varies widely according to the reported cases in the literature. In a study from Iran the age varied from 4 to 36 months [11]. In a study of 222 children from Finland, Kröger and associates showed an age range of 0.25 to 5.7 years [3] and in a similar study the mean age was reported to be 11 months [12].

The diagnosis of osteitis after BCG vaccination in our case was established according to the criteria proposed by Foucard and Hjelmstedt in 1971 which include BCG vaccination in the neonatal period, period of less than 4 years between vaccination and symptom onset, no contact between the child and any adults with tuberculosis, a consistent clinical profile, and histopathology suggestive of tuberculosis [13]. Our case developed a bony lesion in the distal radius which is very rare. Tuberculosis of the hand and wrist is the rarest osteoarticular localization. It represents 2 to 4% of all the localizations of the musculoskeletal system [14].

In our case a prompt diagnosis of tuberculous osteitis of the radius was not made; again this is not surprising as the diagnosis is usually delayed because the lesions may be overlooked due to their rarity, the symptoms tend to develop slowly, and the primary course is fairly benign [10]. Timely diagnosis of BCG osteitis is important since antituberculous therapy is effective when initiated early in the course of disease.

The case described here had a normal CXR; only approximately one third of patients with tuberculosis of the bone have pulmonary involvement, making chest X-ray screening less useful [15].

Treatment of BCG osteitis usually consists of both surgical intervention and antituberculous medication. However, pyrazinamide cannot be used because BCG is resistant to it [10]. The case described here received surgical evacuation as well as antituberculous treatment for 1 year without pyrazinamide with complete healing at follow-up visits. There was no demonstrable difference in the length of both forearms. According to the reported cases in the literature, the long-term evolution of most patients is favorable, and bone sequelae or growth deficit are described in only 3% of the cases [3].

## Conclusions

BCG osteitis, although rare, should be considered a possible complication of the BCG vaccination, and early diagnosis and treatment of this complication are necessary. Osteitis that has a fairly benign course in children should raise suspicion of a tuberculous origin, particularly if the disease does not respond favorably to conventional antibiotics. Despite this rare complication of the BCG vaccine, the use of the vaccine should be maintained in countries with a high incidence of tuberculosis, Sudan is no exception.

## Abbreviations

BCG: Bacillus Calmette–Guérin
CRP: C-reactive protein
CXR: Chest X-ray
ESR: Erythrocyte sedimentation rate
